# The effect of the promiscuity stereotype on opposition to gay rights

**DOI:** 10.1371/journal.pone.0178534

**Published:** 2017-07-13

**Authors:** David Pinsof, Martie G. Haselton

**Affiliations:** Department of Psychology, University of California Los Angeles, Los Angeles, CA, United States of America; Macquarie University, AUSTRALIA

## Abstract

Opposition to gay rights is prevalent in countries around the world. Recent correlational research suggests that opposition to gay rights may be driven by an interaction between one’s own short-term mating orientation (i.e. willingness to engage in casual sex) and representations of gay people as sexually promiscuous. Here, we experimentally manipulated representations of gay men by randomly assigning participants to read one of two versions of a fictitious newspaper article, one of which contained faux scientific evidence confirming the stereotype that gay men are promiscuous, and the other containing faux scientific evidence refuting the stereotype. We found that the manipulation interacted with short-term mating orientation (STMO) to predict opposition to gay rights, such that low-STMO individuals (i.e. more averse to casual sex) exhibited more support for gay rights when assigned to read the stereotype-refuting article compared to the stereotype-confirming article, whereas high-STMO individuals (i.e. less averse to casual sex) were not significantly influenced by the manipulation. We discuss the implications of these findings for the study of antigay attitudes, as well as for recent societal changes in acceptance of homosexuality.

## Introduction

Despite recent trends towards greater acceptance of homosexuality in the United States, opposition to gay adoption and gay marriage are still common, with 39% and 35% of Americans opposed, respectively [1, 2]. Research suggests that the strongest opponents of gay rights tend to be the strongest supporters of marital commitment and family values, and the strongest opponents of divorce and family breakdown [3–6]. Since homosexual relationships do not preclude childrearing or lifelong commitment, these findings are difficult to explain. One might expect supporters of an institution or a way of life to be the most enthusiastic about making it available to more individuals; yet the opposite appears to be the case.

One possible solution to this puzzle relates to differences in *mating strategies* between social liberals and social conservatives. For instance, research has revealed that, relative to social liberals, social conservatives exhibit lower short-term mating orientation (STMO—i.e. interest in casual sex [7, 8]), lower numbers of lifetime sexual partners [9, 10], higher rates of marriage [11], younger ages of childbirth [12], more traditional family structures [13], and larger family sizes [14]. Evolutionary psychologists have theorized that these differences in mating strategies may create conflicts of interest, causing individuals to support the policies and social norms that facilitate their mating strategy [15, 16].

For instance, individuals pursuing the mating strategies typical of social conservatives may be more motivated to condemn sexual promiscuity than their more socially liberal counterparts. For women pursuing this strategy, having a larger number of children at a younger age cannot be as easily achieved without the commitment and financial support of a male-breadwinner. But this leaves women more vulnerable to abandonment: if the relationship dissolves, they are forced to care for a large number of children without adequate resources or career experience. For men, investing greater resources in a larger number of offspring carries opportunity costs (i.e. foregoing short-term mating opportunities) and the possibility of cuckoldry (i.e. diverting time and energy supporting another man’s offspring). These two, central risks—i.e. cuckoldry (for men) and abandonment (for women)—may be larger in environments with widespread sexual promiscuity, because such environments are rife with temptations for individuals to stray from long-term relationships. Thus, men and women pursuing the mating strategies typical of social conservatives may be especially concerned about the societal prevalence of sexual promiscuity.

This concern might translate into a *moral heuristic* [17, 18] that leads to condemnation of promiscuity or any activity that is perceived to be associated with promiscuity (e.g. partying, wearing revealing clothes, etc.). For instance, if recreational drugs are mentally associated with promiscuity, then sexually conservative individuals may condemn recreational drugs [19]. If access to abortion and contraception are viewed as facilitating sexual promiscuity, then sexually conservative individuals may likewise oppose abortion [15, 20]. Moreover, if sexually conservative individuals hold implicit or explicit stereotypes of gay people as sexually promiscuous, then they may oppose homosexuality and gay rights [21]. Such opposition may be irrational, because homosexual promiscuity is unlikely to pose a direct threat to heterosexual relationships.

Nevertheless, our evolved psychology may not produce rational attitudes about the kinds of threats (or lack thereof) posed by homosexual promiscuity. Ancestral small-scale societies would have been unlikely to contain large enough aggregations of gay men and women to create the conditions necessary for homosexual promiscuity to occur. For instance, about 2% of individuals in the United States identify as gay or lesbian [22], which implies that an ancestral society of 150 people would have contained about three gay people. The probability of all three of these people belonging to the same sex and age group, much less being attracted to one another, much less recognizing and acting upon these attractions, is low. Moreover, the 2% figure may be an overestimate: evidence indicates that homosexual behavior is significantly less prevalent in hunter gatherer societies compared to agricultural societies, suggesting that homosexual orientation may have emerged as a result of more recent selection pressures associated with the advent of agriculture [23]. It is therefore plausible that homosexual promiscuity was not a reliable feature of ancestral environments, and that human psychology has not evolved to differentiate between threats posed by homosexual vs. heterosexual promiscuity.

Consistent with these ideas, Pinsof and Haselton [21] found that representations of gay people as promiscuous play a powerful role in predicting opposition to gay marriage in the United States, particularly among individuals pursuing a sexually conservative mating strategy (measured by STMO). Specifically, the researchers found that low-STMO individuals exhibit greater opposition to gay marriage than high-STMO individuals, and that this effect is larger among individuals who have stronger mental representations of gay people as promiscuous. Interestingly, both implicit representations (measured using an implicit association test between images of gay couples and words related to promiscuity) and explicit representations (measured using questionnaire items) independently predicted opposition to gay marriage through their interaction with STMO, implying a distinct role for both conscious and unconscious processes. One limitation of this research, however, is that it is correlational and therefore cannot establish that representations of gay people causally interact with STMO to predict opposition to gay marriage. Moreover, the research is limited to attitudes toward gay marriage, and therefore cannot address whether representations of gay people play a broader role in opposition to gay rights and general disapproval of homosexuality.

Here, we expand on this research by experimentally manipulating representations of gay men in order to investigate the possible causal relationship of these representations with opposition to gay rights (e.g. including adoption rights), contingent on variation in mating strategies (measured by STMO). We randomly assigned participants to read one of two fictitious newspaper articles. One version of the article provided faux scientific evidence confirming the stereotype that gay men are promiscuous, whereas the other version of the article provided faux scientific evidence refuting this stereotype. We first sought to test the effectiveness of the newspaper article in manipulating participants’ implicit and explicit representations of gay men as sexually promiscuous. We then sought to test the hypothesis that exposure to the article would influence participants’ opposition to gay rights, and that this effect would be larger among low-STMO individuals than high-STMO individuals.

## Method

Research was approved by the UCLA Office of Research Administration WebIRB, IRB#14–000053.

### Participants

A total of 1,009 participants were recruited using Amazon’s Mechanical Turk. 147 participants were excluded for either failing our attention check or for having more than 10% of their IAT response times below 300 milliseconds (following Greenwald, Nosek, and Banaji [24]; also following the prior study by Pinsof and Haselton [21]). The resulting sample was 862 participants, with 386 men and 476 women. Participants’ age ranged from 18 to 96, with a mean age of 38 (*SD* = 12). Participants’ varied in their relationship status: 27.6% reported being single or casually dating, 22.6% reported being in a relationship or engaged, 43.6% reported being married, and 6.1% reported being divorced or widowed. The majority of participants (91.6%) reported being heterosexual or mostly heterosexual, 4.5% reported being bisexual, and 3.8% reported being homosexual or mostly homosexual. The sample skewed politically liberal, with 51% of participants identifying as at least slightly liberal, 24% identifying as moderate, and 25% identifying as at least slightly conservative. The sample also skewed politically liberal regarding attitudes toward gay rights, with a mean score of 2.5 (*SD =* 1.95) on a 7-point scale, with 1 representing strong support for gay rights and 7 representing strong opposition.

### Materials and procedure

#### Study design

Participants were randomly assigned to either the stereotype-refuting condition or the stereotype-confirming condition. In the stereotype-refuting condition, participants were instructed to read a newspaper article providing scientific evidence (which was fabricated) that gay men are equally promiscuous as straight men. In the stereotype-confirming condition, participants were instructed to read an otherwise identical newspaper article providing scientific evidence (which was also fabricated) that gay men are more promiscuous than straight men. In both conditions, participants were not told that the evidence was fabricated prior to reading the article. In both conditions, the article was entitled “Are Gay Men Promiscuous?” and contained a picture of a gay couple kissing below the title. In the stereotype-confirming condition, the article began with the text: “There is a common stereotype that gay men are promiscuous. But does the stereotype have any truth to it? Scientific evidence suggests the answer is yes.” In the stereotype-refuting condition, this opening paragraph was identical except for the last sentence, which read: “Scientific evidence suggests the answer is no.” At the end of the study, participants were debriefed and notified that the evidence in the article was fabricated.

#### Explicit representations of gay men as promiscuous

Explicit representations were assessed with four items: “Gay men tend to have more sexual partners throughout their lives than straight men,” “Gay men tend to have more casual sex (i.e. ‘one-night stands’) than straight men,” “In general, gay men tend to be less interested in lifelong, romantic commitment than straight men,” “In general, gay men tend to be less interested in settling down and getting married than straight men.” Participants rated their agreement with the statements on a likert scale ranging from 1 (*strongly disagree*) to 7 (*strongly agree*). Cronbach’s α was 0.97.

#### Implicit representations of gay men as promiscuous

Our methodology for measuring implicit representations of gay men as promiscuous was identical to the methodology used by Pinsof and Haselton [21]. We used a customized Implicit Association Test (IAT) to measure mental associations between images of gay male couples and words related to promiscuity. Participants were instructed to categorize five words related to either promiscuity (“casual sex,” “hookup,” “horny,” “one-night stand,” and “lustful”) or monogamy (“married,” “devoted,” “faithful,” “loving,” “matrimony”) and five images of either gay male couples or opposite sex couples. If participants’ response times are faster when categorizing both gay couples and promiscuous words, then the concepts of “gay” and “promiscuous” are thought to be mentally associated at the implicit level [25]. IAT scores represent the mean difference in response times between the two versions of the task—i.e. the version where “gay” and “promiscuous” are paired and the version where “gay” and “monogamous” are paired—in terms of standard deviations. Higher scores on the IAT indicate stronger mental associations between the concepts “gay” and “promiscuous.” For further details on the methodology, see Pinsof and Haselton [21].

#### Opposition to gay rights

Participants rated their agreement with four statements relating to gay marriage [“Marriage is between a man and a woman,’ “Same-sex marriage undermines the meaning of the traditional family,” “I oppose the legalization of same-sex marriage,” and “Same-sex couples should have the same legal rights to get married as heterosexual couples” (reverse coded)], one statement relating to gay adoption [“Same-sex couples should be prevented from adopting children”], one statement relating to gays in the military [“Gay men and women should be allowed to serve openly in the military” (reverse coded)], and two statements relating to general disapproval of homosexuality [“Homosexuality is immoral” and “There is nothing wrong with being gay” (reverse coded)]. Participants rated their agreement with the statements on a likert scale ranging from 1 (*strongly disagree*) to 7 (*strongly agree*). Factor analysis indicated that a common factor explained 82% percent of the variation of these items. Factor loadings ranged from 0.57 (lowest) to 0.90 (highest), and cronbach’s α was 0.96. We therefore averaged all the items to form a composite measure of opposition to gay rights. When we restricted the measure to only include items used by Pinsof and Haselton [21]—which pertained specifically to gay marriage—the results remained essentially the same (see supporting information). Moreover, when we restricted the measure to only include items that were *not* related to same-sex marriage, the results remained essentially the same (see supporting information).

#### Short-term mating orientation (STMO)

Participants rated their agreement with four statements [26]: “Sex without love is OK,” “I can easily imagine myself being comfortable and enjoying ‘casual sex’ with different partners,” “I could easily imagine myself enjoying one night of sex with someone I would never see again,” and “I could enjoy sex with someone I find highly desirable even if that person does not have long-term potential.” Participants rated their agreement with the statements on a likert scale ranging from 1 (*strongly disagree*) to 7 (*strongly agree*). Cronbach’s α was 0.93.

#### Suspiciousness of the authenticity of the article

In order to assess whether or not participants thought the newspaper article was real, participants rated their agreement with three statements: “While I was reading the article, I did *not* believe any of the information in it,” “While I was reading the article, I assumed that the facts were accurate” (reverse coded), “While I was reading the article, I assumed that it was published in a legitimate newspaper” (reverse coded). Cronbach’s α was 0.91.

## Results

All analyses were conducted using SPSS version 23. We first present data on participants’ suspiciousness of the article, followed by tests of the effectiveness of the manipulation in altering representations of gay men as promiscuous. Then, we present multiple regression analyses that provide key tests of our prediction that the experimental manipulation (dummy coded as 1 for the stereotype-confirming condition and 0 for the stereotype-refuting condition) would interact with short-term mating orientation to predict opposition to gay rights. We first ran analyses with no exclusion criteria (other than those listed above), followed by moderate exclusion criteria and strict exclusion criteria (see following section).

### Suspiciousness of the authenticity of the article

The mean suspiciousness rating was 2.97 (*SD* = 1.8). This value is just below the midpoint of the scale, with a score of 7 indicating strong disagreement that the article was authentic. A substantial number of participants were highly suspicious of the article: 42 participants (5% of the sample) had a score of 7, and 97 participants had a score of 6 or above (11% of the sample). This level of suspiciousness may be common among Mechanical Turk users due to repeated exposure to similar types of manipulations [27]. We were concerned that data from these participants would be less reliable. Accordingly, in the following analyses we have implemented varying levels of exclusion criteria based on participants’ suspiciousness of the article. We define moderate exclusion criteria as removing participants with a score of 7 (42 participants; 5% of sample), and we define strict exclusion criteria as removing participants with a score of 6 or above (97 participants; 11% of the sample). We present results with the full sample for comparison. We also conducted tests of our key predictions controlling for article suspiciousness as a continuous measure, which yielded similar results (data in S1 File).

Examining the full sample, suspiciousness of the article was negatively correlated with opposition to gay rights in the stereotype-confirming condition (r = -0.34, p < .0001) and positively correlated with opposition to gay rights in the stereotype-refuting condition (r = .31, p < .0001). Suspiciousness ratings were higher for the stereotype-confirming article (*M =* 3.35, *SD =* 1.90) than the stereotype-refuting article (*M =* 2.57, *SD =* 1.62), *t* (878) = 6.63, 95% CI of the difference = [0.56, 1.02], p < .0001. This difference may have been due to the fact that our sample skewed pro-gay rights (see section on participant characteristics). For the full sample, the mean suspiciousness rating was 2.97 (*SD* = 1.80); under moderate exclusion criteria, the mean was 2.76 (*SD* = 1.60); and under strict exclusion criteria, the mean was 2.52 (*SD* = 1.35).

### Associations between age, gender, STMO, and opposition to gay rights

For the following correlations, we used data from the full sample. We found a significant correlation between and STMO and opposition to gay rights (r = -0.40, p < .0001), such that low-STMO individuals exhibited stronger opposition to gay rights than high-STMO individuals. Age was significantly (albeit weakly) associated with STMO (r = -0.11, p < .01), with older individuals exhibiting lower STMO than younger individuals. Age was also significantly associated with opposition to gay rights (r = 0.17, p < .0001), with older individuals exhibiting stronger opposition to gay rights than younger individuals. Women exhibited lower STMO scores (*M =* 2.91, *SD =* 1.77) than men (*M =* 4.46, *SD =* 2.05), *t* (878) = -12.02, 95% CI of the difference = [-1.80, -1.30], p < .0001. However, we found no significant effect of gender on opposition to gay rights, *t* (878) = -0.83, 95% CI of the difference = [-0.37, 0.15], p = .41.

### Manipulation check

Examining data from the full sample, participants assigned to the stereotype-confirming condition exhibited stronger explicit representations of gay men as promiscuous (*M* = 5.0, *SD* = 1.66) than participants assigned to the stereotype-refuting condition (*M* = 2.3, *SD* = 1.47), *t* (860) = -26.40, 95% CI of the difference = [-2.74, -2.31], p < .0001. As an additional test of the manipulation’s effectiveness, we examined differences in IAT scores, which are less susceptible to conscious control (and perhaps less vulnerable to demand characteristics). Examining data from the full sample, participants assigned to the stereotype-confirming condition exhibited higher IAT scores (*M* = 0.58, *SD* = .43) than participants assigned to the stereotype-refuting condition (*M* = 0.43, *SD* = .41), *t* (860) = -5.05, 95% CI of the difference = [-0.20, -0.08], p < .0001.

### Interaction between the experimental condition and STMO

**Fig 1:**
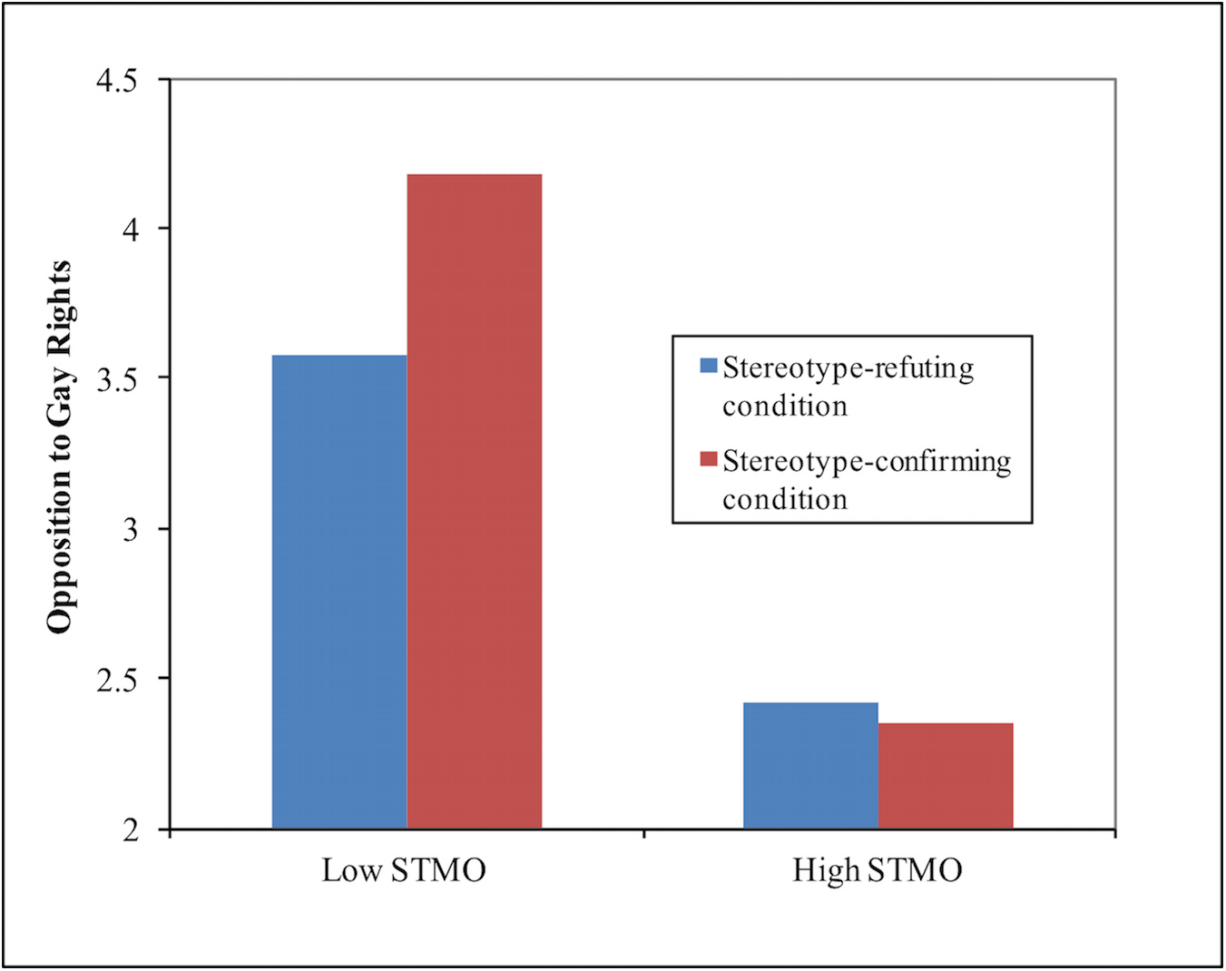
Effect of experimental condition (red vs. blue bars) on opposition to gay rights (y axis) at STMO scores one standard deviation below the mean (low STMO) and at STMO scores one standard deviation above the mean (high STMO).

Consistent with our predictions, we found a significant two-way interaction between the experimental condition and STMO in predicting opposition to gay rights in the full sample (*b* = -.17, SE = .06, 95% CI = [-0.29, -0.05], p < .01). The size of this interaction was similar under moderate exclusion criteria (*b* = -.15, SE = .06, 95% CI = [-0.27, -0.03], p < .01), and under strict exclusion criteria (*b* = -.17, SE = .06, 95% CI = [-0.29, -0.05], p < .0001). Simple slopes tests revealed that, among individuals with STMO scores at one standard deviation below the mean (i.e. low STMO), there was a significant effect of the manipulation on opposition to gay rights in the full sample (*b =* 0.61, SE = .06, 95% CI = [0.50, 0.73], p < .0001). This effect was similar under moderate exclusion criteria (*b =* 0.65, SE = .06, 95% CI = [0.53, 0.77], p < .0001), and slightly larger under strict exclusion criteria (*b =* 0.77, SE = .06, 95% CI = [0.65, 0.89], p < .0001). Among individuals with STMO scores at one standard deviation above the mean (i.e. high STMO), there was no significant effect of the experimental condition on opposition to gay rights in the full sample (*b* = -0.08, SE = .06, 95% CI = [-0.20, 0.04], p = 0.06). This effect also failed to reach significance under moderate exclusion criteria (*b* = 0.04, SE = .06, 95% CI = [-0.06, 0.16], p = 0.32), however the effect became significant (though relatively small) under strict exclusion criteria (*b* = 0.10, SE = .05, 95% CI = [0.01, 0.20], p < .05). For a graph of the interaction using data from the full sample, see Fig 1. In addition, we ran a regression model where we controlled for suspiciousness of the article, and the pattern of results remained essentially the same (data in S1 File).

## Discussion

The results support the hypothesis that representations of gay men as promiscuous interact with mating strategies to predict opposition to gay rights. By presenting participants with contrasting versions of a fictitious newspaper article, we were able to successfully manipulate both implicit and explicit representations of gay men as promiscuous. Consistent with predictions, our experimental manipulation interacted with STMO to predict opposition to gay rights. Specifically, low-STMO participants exhibited more support for gay rights in the stereotype-refuting condition than in the stereotype-confirming condition, whereas high-STMO participants exhibited no difference between the two conditions (though we found a relatively small effect among high-STMO individuals under strict exclusion criteria).

Notably, we were able to influence opposition to gay rights solely by manipulating representations of gay men, as opposed to manipulating representations of both gay men and lesbians. Whether representations of gay men generalize to lesbians, or whether representations of gay men are sufficient to drive opposition to gay rights is a question for future research. We note, however, that representations of lesbians as promiscuous appear to be quite common at the implicit level (mean IAT score = 0.62, see Pinsof & Haselton, [21]), and we find it plausible that such representations could also play a role in opposition to gay rights.

There are, of course, limitations of this research. Our design did not allow us to investigate the temporal duration of the effect of the experimental condition on opposition to gay rights. Future research using longitudinal designs might investigate the temporal robustness of our effects. Moreover, it was not possible for us to determine whether it was STMO in particular—relative to other aspects of mating strategies or their covariates—that caused people to condition their attitudes toward gay rights on representations of gay people as promiscuous. Future research might attempt to experimentally manipulate STMO (e.g. [28]), or examine the effects of STMO when controlling for other relevant variables (e.g. disgust sensitivity, political conservatism [21]).

Our results may have important implications for the study of antigay attitudes. Prior research indicates that antigay attitudes are associated with higher religiosity [29], higher disgust sensitivity [30], and a lower frequency of contact with homosexuals [31]. The ideas guiding our research may provide a parsimonious explanation for all three of these relationships. First, recent research suggests that the primary function of religious institutions across cultures is to facilitate sexually conservative mating strategies—as distinct from promoting other kinds of nonsexual moral concerns [8, 16]. Thus, to the extent that homosexuality is viewed as antithetical to the mating strategies promoted by religious institutions, religious individuals may be especially inclined to disapprove of homosexuality and oppose gay rights. Second, higher disgust sensitivity in the sexual domain is related to more sexually restricted (i.e. low STMO) mating strategies and may represent the affective component of such mating strategies [32]. Thus, disgust sensitivity may be indirectly related to antigay attitudes by virtue of the role it plays in facilitating sexually restricted mating strategies [7]. Third, limited contact with homosexuals may increase the extent to which individuals rely on the promiscuity stereotype in judging same-sex romantic relationships. Increased contact with homosexuals may therefore provide stereotype-refuting information that reduces antigay prejudice. If this is the case, one might expect the relationship between homosexual contact and antigay prejudice to be particularly pronounced among low-STMO individuals, and to be mediated by representations of gay people as promiscuous.

Our findings are consistent with an emerging body of research on the role of “value conflicts” in generating intergroup prejudice [6, 33]. However, our approach expands on this research by specifying where these values come from, why they contain the contents they do, why some individuals hold them while others do not, and why they create conflict in particular, as opposed to mere confusion or unfamiliarity [8, 20]. Moreover, our approach leads to testable predictions about when, and in which circumstances, sexually conservative values will emerge (e.g. in ecological contexts favoring long-term mating strategies), potentially shedding light on variation in values across cultures and over time.

For instance, in the United States, acceptance of homosexuality could have emerged as a result of short-term mating strategies becoming more prevalent, and as new cultural values evolved to facilitate these mating strategies. Indeed, research indicates that attitudes toward non-marital sexual behavior have become more lenient in recent decades [34], possibly as a result of ecological factors such as increasing female economic independence [35], decreased risk of sexually transmitted diseases [36], and/or changing sex ratios among particular demographic groups [37]. Whether or not changes in mating strategies coincided with or temporally preceded changes in acceptance of homosexuality is a question for future research.

Another potential cause of increasing acceptance of homosexuality may have been changes in people’s representations of same-sex relationships as sexually promiscuous. For instance, early state legalizations of gay marriage could have led to increases in media depictions of committed, family-oriented gay couples, and this could have initiated a positive feedback loop leading to greater acceptance of homosexuality and further increases in state legalizations of gay marriage. To what extent media depictions of committed gay couples are capable of altering representations of gay men and lesbians—and thereby increasing support for gay rights—is an additional question for future research.

One implication of the ideas guiding this research is that antigay attitudes are far from inevitable. If antigay attitudes are contingent on specific mating strategies interacting with specific mental representations, both of which may be capable of undergoing rapid change, then antigay attitudes may be more of a product of cultural and ecological circumstances than an immutable feature of human nature. Thus, the ideas guiding this research may provide a reason for gay rights activists to be optimistic about the continuing decline of opposition to gay rights.

## Supporting information

S1 File Supplementary analyses. Analyses pertaining to opposition to gay marriage as the dependent variable, opposition to gay rights (but not gay marriage) as the dependent variable, and analyses controlling for suspiciousness of the article.
(DOCX)

## References

[1] Doherty C (2015) Negative views of Supreme Court at record high, driven by Republican dissatisfaction. Retrieved from the Pew Research Center Web site: http://www.peoplepress.org/2015/07/29/negative-views-of-supreme-court-atrecord-high-driven-by-republican-dissatisfaction/2/

[2] Swift A (2015) Most Americans Say Same-Sex Couples Entitled to Adopt. Retrieved from the Gallup web site: http://www.gallup.com/poll/170801/americans-say-sex-couples-entitled-adopt.aspx

[3] Brumbaugh SM, Sanchez LA, Nock SL, Wright JD (2008) Attitudes toward gay marriage in states undergoing marriage law transformation. J Marriage Fam 70: 345–359.

[4] McVeigh R, Maria-Elena DD (2009) Voting to ban same-sex marriage: Interests, values, and communities. Am Sociol Rev 74: 891–915.

[5] Craig SC, Martinez MD, Kane JG (2005) Core values, value conflict, and citizens’ ambivalence about gay rights. Polit Res Quart 58: 5–17.

[6] Reyna C, Wetherell G, Yantis C, Brandt MJ (2014) Attributions for sexual orientation vs. stereotypes: how beliefs about value violations account for attribution effects on anti‐gay discrimination. J Appl Soc Psychol 44: 289–302.

[7] Tybur JM, Inbar Y, Güler E, Molho C (2015) Is the relationship between pathogen avoidance and ideological conservatism explained by sexual strategies?. Evol Hum Behav 36: 489–497.

[8] Rowatt WC, Schmitt DP (2003) Associations between religious orientation and varieties of sexual experience. J Sci Stud Relig 42: 455–465.

[9] Brody S, Rau H, Führer N, Hillebrand H, Rüdiger D, Braun M (1996) Traditional ideology as an inhibitor of sexual behavior. J Psychol 130: 615–626.

[10] Weeden J, Cohen AB, Kenrick DT (2008) Religious attendance as reproductive support. Evol Hum Behav 29: 327–334. doi: 10.1016/j.evolhumbehav.2008.03.004 21874105 10.1016/j.evolhumbehav.2008.03.004 PMC3161130

[11] Smith TW (2008) Changes in family structure, family values, and politics, 1972–2006. GSS Social Change Report No. 53. Chicago: National Opinion Research Center, University of Chicago. Retrieved from http://publicdata.norc.org:41000/gss/ documents/scrt/sc53%20changes%20in%20family%20structure,% 20family%20values,%20and%20politics,%201972-2006.pdf

[12] Cahn N, Carbone J (2010) Red families v. blue families: Legal polarization and the creation of culture Oxford University Press.

[13] Monson RA, Mertens JB (2011) All in the Family: Red States, Blue States, and Postmodern Family Patterns, 2000 and 2004. Sociol Quart 52: 244–267.

[14] Hayford SR, Morgan SP (2008) Religiosity and fertility in the United States: The role of fertility intentions. Soc Forces 86: 1163–1188. doi: 10.1353/sof.0.0000 19672317 10.1353/sof.0.0000 PMC2723861

[15] Weeden J, Kurzban R (2014) *The Hidden Agenda of the Political Mind*: *How Self-interest Shapes Our Opinions and Why We Won't Admit it*. Princeton University Press.

[16] Weeden J, Kurzban R (2013) What predicts religiosity? A multinational analysis of reproductive and cooperative morals. Evol Hum Behav 34: 440–445.

[17] Sunstein CR (2005) Moral heuristics. Behav Brain Sci 28: 531–541. doi: 10.1017/S0140525X05000099 16209802 10.1017/S0140525X05000099

[18] Cosmides L, Tooby J (2006) Evolutionary psychology, moral heuristics, and the law In Heuristics and the Law. MIT Press; Cambridge, MA: 2006 pp. 182–212.

[19] Kurzban R, Dukes A, Weeden J (2010) Sex, drugs and moral goals: Reproductive strategies and views about recreational drugs. P R Soc B 277: 3501–3508. 10.1098/rspb.2010.0608 PMC2982222 20554547

[20] Weeden J (2003) Genetic interests, life histories, and attitudes towards abortion. Unpublished doctoral dissertation, University of Pennsylvania, Philadelphia.

[21] Pinsof D, Haselton MG (2016) The Political Divide Over Same-Sex Marriage: Mating Strategies in Conflict? Psychol Sci 27: 435–442. doi: 10.1177/0956797615621719 26921411 10.1177/0956797615621719

[22] Gates G (2011) How many people are lesbian, gay, bisexual, and transgender? The Williams Institute Retrieved from https://williamsinstitute.law.ucla.edu/wp-content/uploads/Gates-How-Many-People-LGBT-Apr-2011.pdf

[23] Apostolou M (2016) Is Homosexuality more Prevalent in Agropastoral than in Hunting and Gathering Societies? Evidence from the Standard Cross-Cultural Sample. Adapt Hum Behav Physiol 3: 1–10.

[24] Greenwald AG, Nosek BA, Banaji MR (2003) Understanding and using the implicit association test: I. An improved scoring algorithm. J Pers Soc Psychol 85: 197 12916565 10.1037/0022-3514.85.2.197

[25] Greenwald AG, McGhee DE, Schwartz JL (1998) Measuring individual differences in implicit cognition: the implicit association test. J Pers Soc Psychol 74: 1464 9654756 10.1037//0022-3514.74.6.1464

[26] Jackson JJ, Kirkpatrick LA (2007) The structure and measurement of human mating strategies: Toward a multidimensional model of sociosexuality. Evol Hum Behav 28: 382–391.

[27] Stewart N, Ungemach C, Harris AJ, Bartels DM, Newell BR, Paolacci G, Chandler J (2015) The average laboratory samples a population of 7,300 Amazon Mechanical Turk workers. Judgm Decis Mak 10: 479–491.

[28] Moss JH, Maner JK (2016) Biased sex ratios influence fundamental aspects of human mating. Pers Soc Psychol B 42: 72–80. 10.1177/0146167215612744 26498976

[29] Whitley BE Jr (2009) Religiosity and attitudes toward lesbians and gay men: A meta-analysis. Int J Psychol Relig 19: 21–38.

[30] Olatunji BO (2008) Disgust, scrupulosity and conservative attitudes about sex: Evidence for a mediational model of homophobia. J Res Pers 42: 1364–1369.

[31] Hodson G, Harry H, Mitchell A (2009) Independent benefits of contact and friendship on attitudes toward homosexuals among authoritarians and highly identified heterosexuals. Eur J Soc Psychol 39: 509–525.

[32] Al-Shawaf L, Lewis DM, Buss DM (2015) Disgust and mating strategy. Evolution and Human Behavior, 36: 199–205.

[33] Chambers JR, Schlenker BR, Collisson B (2013) Ideology and prejudice: The role of value conflicts. Psychol Sci 24: 140–149. doi: 10.1177/0956797612447820 23287021 10.1177/0956797612447820

[34] Smith TW (1994) Attitudes toward sexual permissiveness: Trends, correlates, and behavioral connections. Sexuality across the life course: 63–97.

[35] Price ME, Pound N, Scott IM (2014) Female economic dependence and the morality of promiscuity. Arch Sex Behav 43: 1289–1301.# doi: 10.1007/s10508-014-0320-4 24961579 10.1007/s10508-014-0320-4 PMC4161927

[36] Francis AM (2013) The wages of sin: How the discovery of penicillin reshaped modern sexuality. Arch Sex Behav 42: 5–13. doi: 10.1007/s10508-012-0018-4 23054260 10.1007/s10508-012-0018-4

[37] Pedersen FA (1991) Secular trends in human sex ratios. Hum Nature 2: 271–291. 24222281 10.1007/BF02692189

