# Supplementary material for: The effect of the promiscuity stereotype on opposition to gay rights
Source: PLoS One. 2017 Jul 13;12(7):e0178534. doi: 10.1371/journal.pone.0178534 (PMC5509117; doi:10.1371/journal.pone.0178534)
Supplement: S1 File — Analyses pertaining to opposition to gay marriage as the dependent variable, opposition to gay rights (but not gay marriage) as the dependent variable, and analyses controlling for suspiciousness of the article. (DOCX) [file pone.0178534.s001.docx]

***Interaction between the experimental condition and STMO in predicting opposition to gay marriage.*** For these analyses, we used a composite of the four items on attitudes toward gay marriage (cronbach’s α = 0.96) as our dependent measure. We found a significant two-way interaction between the experimental condition and STMO in predicting opposition to gay marriage (*b* = -.15, SE = .06, 95% CI = [-0.27, -0.03], p < .05). The size of this interaction was similar under moderate exclusion criteria (*b* = -.13, SE = .07, 95% CI = [-0.25, 0.00], p = .06), and under strict exclusion criteria (*b* = -.15, SE = .07, 95% CI = [-0.28, -0.01], p < .05). Simple slopes tests revealed that, among individuals with STMO scores at one standard deviation below the mean (i.e. low STMO), there was a significant effect of the manipulation on opposition to gay marriage in the full sample (*b =* 0.58*,* SE = .06, 95% CI = [0.46, 0.70], p < .0001). This effect was similar under moderate exclusion criteria (*b =* 0.62*,* SE = .06, 95% CI = [0.50, 0.74], p < .0001), and slightly larger under strict exclusion criteria (*b =* 0.75*,* SE = .06, 95% CI = [0.63, 0.87], p < .0001). Among individuals with STMO scores at one standard deviation above the mean (i.e. high STMO), there was no significant effect of the experimental condition on opposition to gay marriage in the full sample (*b* = -0.03, SE = .06, 95% CI = [-0.15, 0.09], p = 0.43). This effect became positive and significant (though relatively small) under moderate exclusion criteria (*b* = 0.11, SE = .05, 95% CI = [0.01, 0.21], p = < .05) and remained similar under strict exclusion criteria (*b* = 0.17, SE = .06, 95% CI = [0.05, 0.29], p < .001).

***Interaction between the experimental condition and STMO in predicting opposition to gay rights and homosexuality (but* not *gay marriage).*** For these analyses, we used a composite of the four items that were unrelated to gay marriage—i.e. gay adoption, gays in the military, and disapproval of homosexuality (cronbach’s α = 0.93)—as our dependent measure. We found a significant two-way interaction between the experimental condition and STMO in predicting opposition to gay rights and homosexuality (*b* = -.19, SE = .06, 95% CI = [-0.30, -.08], p < .01). The size of this interaction was similar under moderate exclusion criteria (*b* = -.18, SE = .06, 95% CI = [--0.29, -0.07], p < .01), and under strict exclusion criteria (*b* = -.19, SE = .06, 95% CI = [-0.31, -.07], p < .01). Simple slopes tests revealed that, among individuals with STMO scores at one standard deviation below the mean (i.e. low STMO), there was a significant positive effect of the manipulation on opposition to gay rights and homosexuality in the full sample (*b =* 0.63*,* SE = .06, 95% CI = [0.51, 0.75], p < .0001). This effect was similar under moderate exclusion criteria (*b =* 0.68*,* SE = .06, 95% CI = [0.56, 0.80], p < .0001), and slightly larger under strict exclusion criteria (*b =* 0.79*,* SE = .06, 95% CI = [0.67, 0.91], p < .0001). Among individuals with STMO scores at one standard deviation above the mean (i.e. high STMO), there was a small but significant *negative* effect of the experimental condition on opposition to gay rights and homosexuality in the full sample (*b* = -0.12, SE = .05, 95% CI = [-0.22, -0.02], p < .05), with individuals in the stereotype-confirming condition expressing slightly less opposition to gay rights and homosexuality than individuals in the stereotype-refuting condition. This effect, however, dropped below significance under moderate exclusion criteria (*b* = -.02, SE = .06, 95% CI = [-0.14, 0.10], p = 0.64) and remained nonsignificant under strict exclusion criteria (*b* = 0.03, SE = .06, 95% CI = [-0.11, 0.15], p = 0.60).

***Interaction between experimental condition and STMO in predicting opposition to gay rights (using all items), controlling for suspiciousness of the article.*** Using data from the full sample, we found a significant two-way interaction between the experimental condition and STMO in predicting opposition to gay rights (*b* = -.17, SE = .06, 95% CI = [-0.29, -0.05], p < .01). Simple slopes tests revealed that, among individuals with STMO scores at one standard deviation below the mean (i.e. low STMO), there was a significant effect of the manipulation on opposition to gay rights (*b =* 0.61*,* SE = .06, 95% CI = [0.50, 0.73], p < .0001). Among individuals with STMO scores at one standard deviation above the mean (i.e. high STMO), there was no significant effect of the experimental condition on opposition to gay rights (*b* = -0.08, SE = .06, 95% CI = [-0.20, 0.04], p = 0.06).
